# Erythema induratum of Bazin as an indicative manifestation of cavitary tuberculosis in an adolescent: a case report

**DOI:** 10.1186/s12879-021-06454-4

**Published:** 2021-08-03

**Authors:** Kun Yang, Tingying Li, Xiaomei Zhu, Yun Zou, Dongxian Liu

**Affiliations:** Department of Dermatology and Venereology, Tongji Hospital, Tongji Medical College, Huazhong University of Science and Technology, 1095 Jiefang Avenue, Wuhan, 430030 China

**Keywords:** Erythema induratum of Bazin, Cavitary tuberculosis, Adolescents, Case report

## Abstract

**Background:**

Erythema induratum of Bazin (EIB) is a rare cutaneous manifestation of tuberculosis (TB) that frequently occurs in middle-aged women. The clinical manifestations of EIB can mimic various skin diseases, easily leading to misdiagnosis or underdiagnosis. We report a case of a 16-year-old Chinese girl who initially presented with EIB and was subsequently diagnosed with cavitary TB.

**Case presentation:**

The patient is a 16-year-old Chinese girl who presented with clusters of erythematous subcutaneous nodules on the posterior part of her right lower leg matching the clinical manifestations of EIB but was otherwise asymptomatic. The patient was subsequently diagnosed with cavitary TB on the basis of a positive result from a T-SPOT.TB test and the observation of cavitary lung lesions in chest computed tomography scanning. A good clinical response was observed in the patient after the initial phase of a standard 6-month antibiotic regimen.

**Conclusions:**

Adolescents infected with *Mycobacterium tuberculosis* frequently present atypical symptoms. Cutaneous manifestations of tuberculosis in adolescents can be considered good clinical indicators to predict the underlying disease. Strong clinical suspicion is required for a prompt diagnosis in adolescents with EIB.

## Background

Erythema induratum of Bazin (EIB) is a rare nodular tuberculid that predominately occurs on the legs of young or middle-aged women [1, 2]. It is characterized by recurrent clusters of erythematous, tender, subcutaneous nodules with or without ulceration. Histopathologically, EIB presents as panniculitis with varying combinations of granulomatous inflammation, primary vasculitis, and necrosis. *Mycobacterium tuberculosis (M. TB)* has been shown to be etiologically linked to EIB skin lesions, which are caused by a tuberculin hypersensitivity reaction. However, not all patients are positive for *M. TB* DNA in skin biopsies [3]. Moreover, the clinical manifestations of EIB can mimic various skin diseases, such as nodular vasculitis, inflammatory panniculitis, erythema nodosum, and polyarteritis nodosa. These characteristics can easily lead to misdiagnosis or underdiagnosis, preventing prompt treatment.

Adolescents are a key subpopulation of TB patients [4]. However, the diagnosis of adolescent tuberculosis remains a challenge. One reason for this is that the clinical symptoms of adolescent tuberculosis are complicated and atypical due to increased immune competence and hormonal changes in adolescents [5]. Adolescent tuberculosis can present as adult-type tuberculosis accompanied by cavitation but with atypical manifestations, which makes diagnosis challenging if chest radiography is not performed or is not readily available [6]. Adolescents play a vital role in community TB spread and are the main target population for reducing the TB burden. Therefore, it is crucial to accurately diagnose and initiate timely treatment for adolescent tuberculosis. Here, we report a case of a 16-year-old Chinese girl who presented with EIB as an indicator of cavitary TB and who had a favorable response to antituberculosis therapy.

## Case presentation

**Fig. 1:**
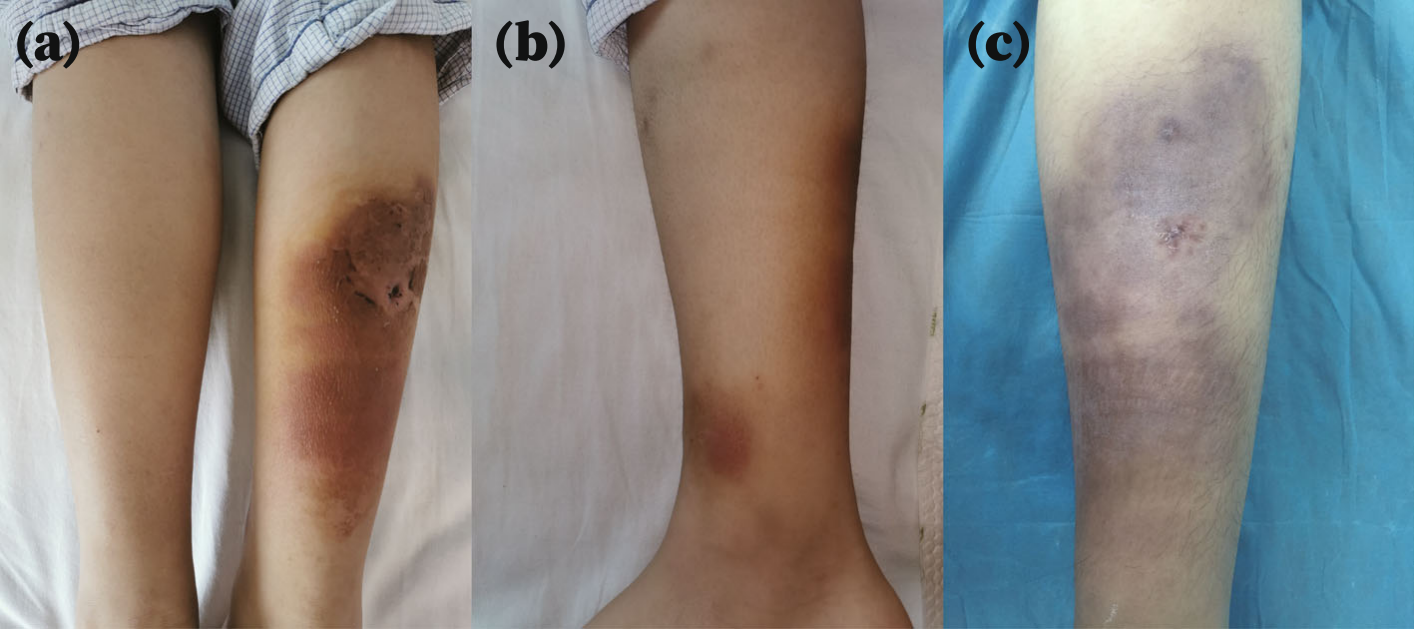
Erythematous nodules on the lower right calf (**a**), near the medial malleolus (**b**) and after an initial 2-month regimen (**c**)

**Fig. 2:**
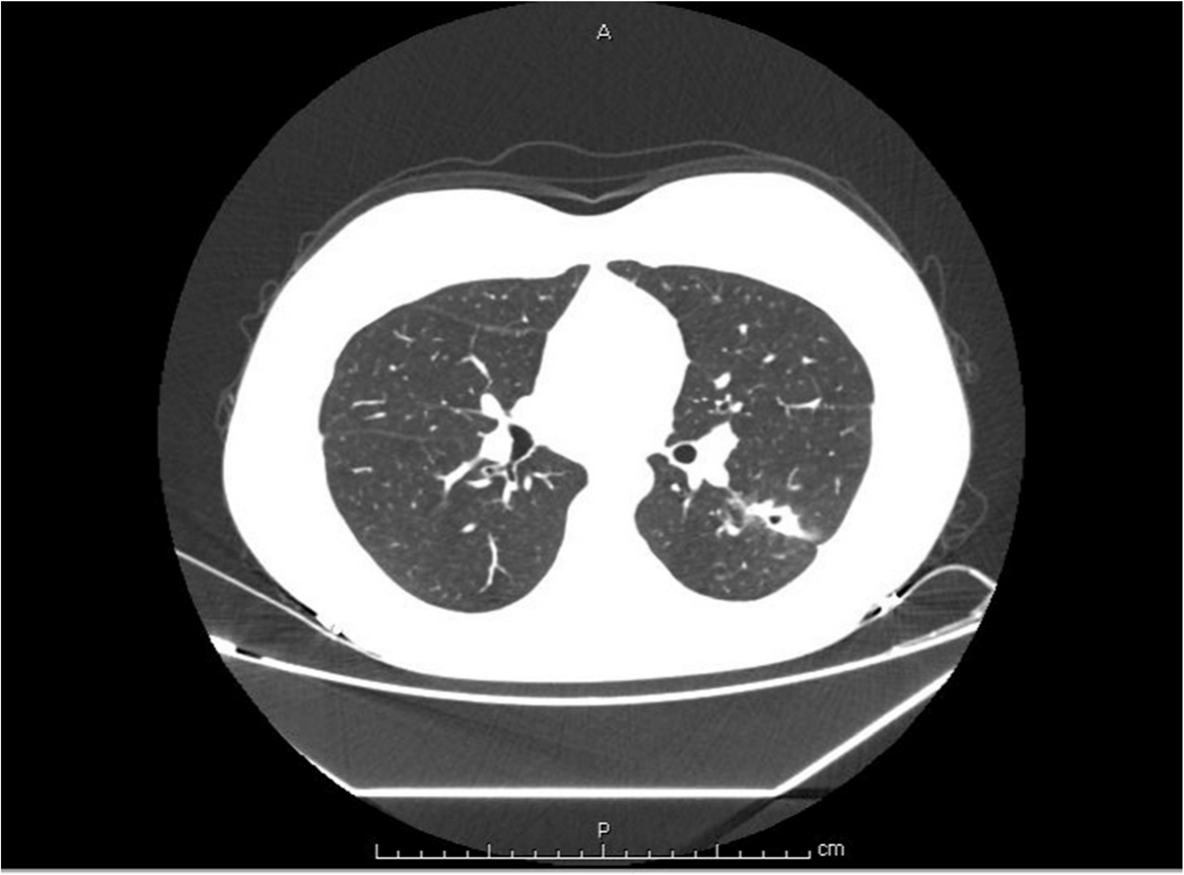
Multiple cavitary lesions and nodules in the lower left lobe were identified on chest computed tomography

**Fig. 3:**
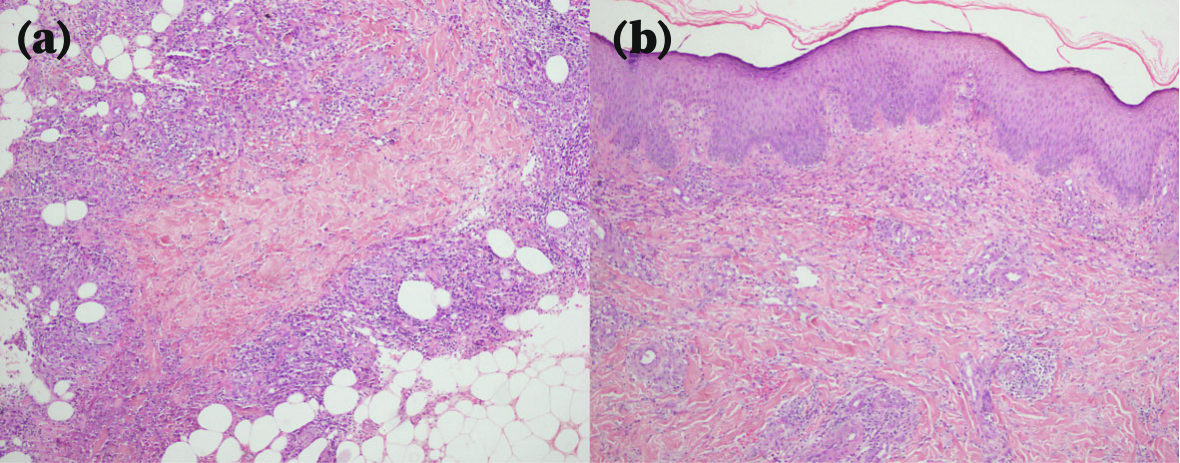
Histopathology of a representative skin biopsy of the leg showing granulomatous septal panniculitis (HE, 100×) (**a**) and small-vessel vasculitis (**b**) (HE, 100×)

A 16-year-old Chinese female presented with a 4-month history of painful erythematous nodules on her right lower leg. She visited a local hospital before referral to our hospital and received antibiotic treatment (sulbactam and cefoperazone) and ichthammol ointment. However, there was no improvement. On physical examination, clusters of small, tender nodules, 1–2 cm in diameter, were palpable on her right posterior calf. The erythema overlying the nodules merged, forming a lesion 10 cm in diameter without ulceration. Right ankle swelling was also observed. A painful erythematous nodule was discovered near the medial malleolus (Fig. 1). The patient had no cough, fever, night sweats, fatigue, or weight loss. She was reported to be previously healthy and not taking any medication. She was vaccinated with the Bacille Calmette-Guerin (BCG) vaccine at birth and had no known exposure to *M. TB* or family history of TB. Laboratory tests, including routine blood tests, erythrocyte sedimentation rate calculation, C-reactive protein detection, and other autoimmunity marker detection, did not reveal abnormalities. However, there was a slight increase in interleukin 6. She was HIV negative. Chest computed tomography (CT) findings revealed multiple cavitary lesions and nodules in the lower left lobe, as well as hyperplastic mediastinal lymph nodes (Fig. 2). A skin punch biopsy was performed, and histopathology showed granulomatous septal panniculitis and small-vessel vasculitis (Fig. 3). The T-SPOT.TB test result was strongly positive. The sputum smear was negative for acid-fast bacilli, as was the sputum culture. The identification of *M. TB* DNA in skin lesions by polymerase chain reaction had not been performed. The patient was diagnosed with cavitary TB. The differential diagnoses in this case included erythema nodosum, sclerosing panniculitis and polyarteritis nodosa. She was put on a six-month regimen that consisted of 2 months of isoniazid, rifampin, ethambutol, and pyrazinamide followed by 4 months of isoniazid, rifampin and ethambutol. Adherence to treatment was assessed every month. After 2 months of intensive therapy, the nodules disappeared, and the erythema turned a dark, reddish-brown color. A significant reduction in cavity size and fewer residual lesions were shown by chest radiography after 6 months of treatment. Adverse and unanticipated events were not observed.

## Discussion and conclusions

Erythema induratum of Bazin, first described by Bazin in 1861, is characterized by chronic, tender, erythematous, indurated subcutaneous nodules on the lower extremities caused by tuberculin hypersensitivity [7]. Historically, the causal relationship between tuberculosis and EIB has been debated due to a lack of etiological evidence because *M. TB* has been isolated from skin lesions only sporadically, and most patients do not present with common symptoms of active tuberculosis [8, 9]. However, with the development of molecular diagnostics, the debate about the etiology ended with the identification of *M. TB* DNA in skin lesions from EIB patients [3].

Currently, EIB is classified as a tuberculid, a type of hematogenous cutaneous tuberculosis (CTB). CTB is rare, comprising 1–1.5% of all extra-pulmonary tuberculosis manifestations which accounts for 8.4–13.7% of all tuberculosis cases [10]. EIB has been reported as the most common form of CTB in Asia, including mainland China, Hong Kong and Japan, and in South America [11–14]. Although both females and males can be affected by EIB, approximately 80% of patients with EIB are females. The age of onset ranges from early childhood to late adulthood. EIB has been frequently reported to have the highest prevalence in middle-aged patients [9, 15, 16]. However, according to a study with a larger sample size, younger age groups predominate among individuals with EIB in China [11]. Our patient was a 16-year-old Chinese girl, and similar cases in adolescents have previously been reported [17, 18]. Therefore, awareness should be increased, and a detailed examination should be performed to ensure a correct diagnosis in young patients with suspected EIB.

Adolescents (aged 10–19 years) have a notably higher risk of tuberculosis infection and disease progression than children in middle childhood (5–9 years), partly because of shifting social contact patterns and immunological or hormonal changes associated with puberty [4, 5]. The diagnosis of tuberculosis can be difficult in adolescents with paucibacillary TB because *M. TB* cultures and smears are often negative. Moreover, tuberculosis in adolescents is protean and can manifest as extrapulmonary disease. Therefore, diagnosis is often incorrect or late due to a lack of clinical suspicion. Our patient presented with EIB as the initial manifestation of TB and was otherwise asymptomatic. If the T-SPOT.TB test and CT scans were not performed, cavitary TB would not have been found. It has been reported that more than 50% of adolescents present with adult-type pulmonary TB characterized by cavitation, which is increasing in frequency in girls aged approximately 14 years and boys aged approximately 15 years [5]. The reason for the high incidence of adult-type pulmonary TB with cavitation during adolescence is that the acquired cellular immune response serves as a double-edged sword capable of disease containment as well as the development of parenchymal cavities due to excessive tissue necrosis [19]. Cavitation greatly increases the risk of person-to-person transmission [20]. In 2019, 396,000 TB cases among children and adolescents aged 10–19 years were reported, equivalent to 10% of the total cases in the 10 countries with the highest TB burdens, of which China is one [21]. EIB accounts for more than 35% of CTB cases in Chinese adolescents [11]. However, no complications of EIB with cavitary TB have been reported. Our case report suggests that EIB could be a screening indicator of underlying TB in adolescents.

The general principles for the management of adolescent tuberculosis should be based on the patient’s overall condition. Our patient presented with EIB but without other typical TB symptoms, and cavitation was observed despite a negative sputum culture result. Therefore, a standard 6-month antibiotic regimen was administered, involving an initial 2-month regimen of isoniazid, rifampicin, pyrazinamide, and ethambutol. A good clinical response was observed in our patient after the initial phase. The nodules on her calf disappeared, and the erythema turned a dark, reddish-brown color. She then took a second antibiotic regimen, which included isoniazid, rifampin and ethambutol, for an additional 4 months. A significant reduction in cavity size and fewer residual lesions were shown by chest radiography. According to TB management guidelines [22, 23] a continued 3-month treatment is being taken, and careful follow-up is being performed.

In conclusion, we report the case of an adolescent presenting with EIB as an initial skin manifestation, leading to the detection of cavitary TB. The clinical manifestations of TB in adolescents could be explained by the maturation of host immunity. This case report highlights the diagnostic value of skin manifestations in adolescents with TB. Strong clinical suspicion is required for a prompt diagnosis of the underlying disease in adolescents with EIB.

### Patient perspective

At first, I was misdiagnosed with panniculitis. A two-week treatment with sulbactam, cefoperazone and ichthammol ointment did not improve the nodules on my leg, which made me feel anxious. Owing to Dr. Liu’s clinical experience and a comprehensive examination, I was finally diagnosed with cavitary TB even without common symptoms of TB. I began to feel confident when the nodules started to disappear within 2 months after I took TB medication. I have completed the 6-month course of standard therapy and returned to the hospital for a reassessment. Dr. Liu advised me to continue to take the medication for 3 months. The whole course of treatment did not place a financial burden on my family thanks to free medications provided by China's national tuberculosis control program.

## Data Availability

Data relating to this study are contained and presented in this document. Other materials are available from the corresponding authors on reasonable request.

## Abbreviations

BCG: Bacille Calmette-Guerin
CT: Chest computed tomography
CTB: Cutaneous tuberculosis
EIB: Erythema induratum of Bazin
TB: Tuberculosis
